# Closed Catheter Drainage for Klebsiella pneumoniae Empyema Necessitans

**DOI:** 10.1155/2023/6668677

**Published:** 2023-10-26

**Authors:** Mohammed Ishaq, Abdullah Bahadi

**Affiliations:** King Saud Medical City, Riyadh, Saudi Arabia

## Abstract

Empyema necessitans is a rare complication of pneumonia, which can be seen more frequently with tuberculosis (TB). In this case report, we include a 47-year-old female with empyema necessitans; closed drainage has been sought as a sole treatment based on the clinical presentation and radiological findings of an empyema necessitans. Scarce evidence with regard to adult empyema necessitans with Klebsiella was found in the literature where adult cases were managed exclusively using combination of intravenous (IV) antibiotic and closed drainage, reporting such case can help guiding management for similar presentation.

## 1. Introduction

Empyema necessitans (also referred to as empyema necessitatis is rare and infrequently reported complication of pneumonia, which involves extension of the empyema and dissecting through chest wall to rest in subcutaneous tissue [1], with limited data on results of management of such cases. Such complications are worrisome for surgeons as empyema extends to involve other parts of the thoracic cavity.

A complication which is commonly associated with tuberculosis patients [2] requires careful management in means of excluding tuberculosis if it was suggested by history, labs, and radiological findings suggestive of TB. Catheter drainage for empyema necessitans without surgery has been practiced in rare cases with astounding success [3, 4].

## 2. Case Report

**Figure 1:**
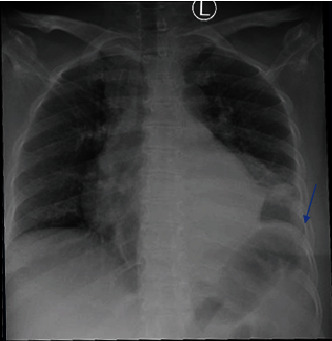
Chest x-ray done with patient presentation to the emergency department showing left lower lobe opacification and obliteration of the costophrenic angle due to pneumonia complicated by parapneumonic effusion (Blue arrow).

A 47-year-old female patient with known case of diabetes mellitus type 2 (uncontrolled) visited the emergency department and was complaining of shortness of breath and subjective fever, night sweat for 7 days, and productive cough that is yellowish in color, and there is no history suggestive of anorexia or weight loss; the patient also complained of left-sided chest swelling increasing in size over 7 days without mention of redness or discharge. On examination, the patient was vitally stable and afebrile. The patient mentioned history of contact with a TB patient. The patient was admitted to the hospital as a case of left-sided pneumonia complicated with pleural effusion based on initial imaging studies that shows obliteration of the left costophrenic angle and left lower lobe opacification (Figure 1), and based on initial labs, she had leukocytosis with white blood cells (WBC) of 17 (10^∗^9/L) and C-reactive protein (CRP) of 257 mg/dL. Kidney function test showed creatinine 50 *μ*mol/L, platelets 445 (10^∗^9/L), alanine transaminase 10.3 U/L, aspartate transaminase 19.5 U/L, and HbA1c 14.8%.

Upon examination, the patient showed bronchial breathing pattern on the left lung specifically the lower lobe and normal breathing sounds in the right lung with no added sounds. A palpable, fluctuant collection is present on the left side of the chest wall, overlaying the 6th to 8th rib, just along the axillary line. The collection exhibits a soft consistency, audible crepitations upon palpation, and retractability of the swelling following reduction. There was no evidence of external skin erythema or pus discharge with completely intact skin.

The treatment for community-acquired pneumonia was started in the emergency department including IV ceftriaxone, IV azithromycin, and IV acetaminophen for pain control. 8 acid-fast bacillus sputum samples with 3 TB polymerase chain reaction (TB-PCR) samples were taken to rule out TB based on patient history that reported negative 3 days later; the patient showed significant improvement with IV antibiotics. Computerized tomography (CT) scan was requested to rule out complicated parapneumonic effusion that could be extending to the lateral chest wall (empyema necessitans).

**Figure 2:**
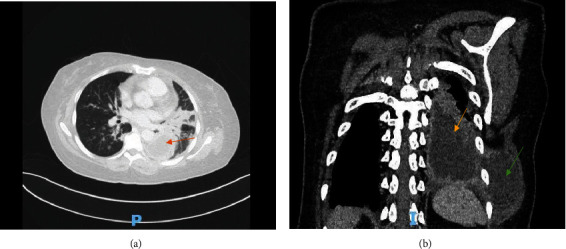
CT scan showing (a) left lower lobe consolidation and atelectatic changes with visualization of pleural collection (orange arrow) and (b) pleural effusion (yellow arrow) in the pleural cavity with extension throughout chest wall (empyema necessitans) (green arrow).

The CT scan (Figure 2) showed patency of trachea and major airways with bilateral ground-glass opacities and consolidation with atelectasis involving the left lower lobe. In addition, a loculated left-sided heterogeneous pleural effusion with internal air foci extends throughout the chest wall with clear area of communication.

Pigtail catheter drainage was inserted in the Interventional Radiology department using ultrasound introducing 12-French (Fr) sized catheter in the 5^th^ intercostal space through chest wall posteriorly through ultrasound guidance.

Initially, 400 cc of yellowish turbid thick foul-smelling fluid was drained in the underwater seal. Daily maintenance of the catheter was assured with flushing 10 mL of normal saline 0.9% through the catheter port twice a day. Additionally, daily massage of the external component was done to drain it through intrathoracic communicating tract; drained fluids were sent for TB-PCR, acid-fast bacillus culture, culture, and sensitivity and pleural fluid analysis for microscopic examination; and the results were negative for Mycobacterium with culture being positive for Klebsiella pneumoniae sensitive to fluoroquinolones. Pleural fluid analysis results are shown in Table 1.

**Figure 3:**
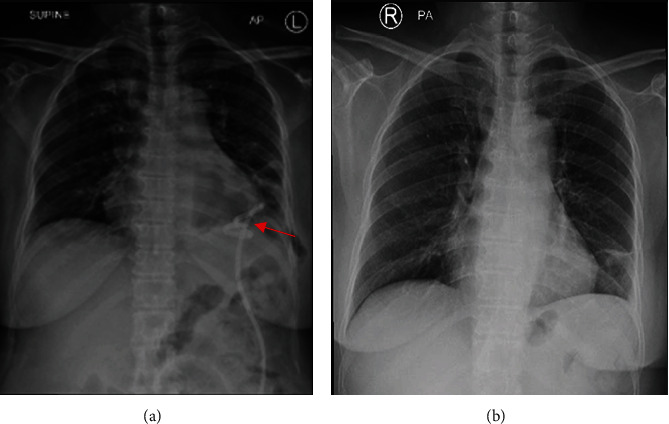
Chest X-ray postinsertion and predischarge. (a) Postinsertion of pigtail catheter in the left thoracic cavity (Red arrow). (b) Chest X-ray showing complete resolution of empyema and remarkable expansion of the left lower lobe.

Complete drainage was achieved in 2 days with remarkable results demonstrated on roentgenogram showing complete resolution of pneumonia and soft tissue swelling (Figure 3(b)); the patient improved remarkably clinically with no palpable soft tissue swelling and was ready for discharge (after a total of 4 days of admission) without the need for further surgical intervention on moxifloxacin for more 14 days based on our infectious disease opinion.

## 3. Discussion

Management of empyema necessitans has diverse means of treatment that to this day was tailored for each case without algorithmic approach. Most cases found in literature were managed using different techniques that included video-assisted thoracoscopy for evacuation of empyema, debridement, and decortication in case of fibrothorax with thick pleura and trapping of the lung [2, 5], incision and drainage [6], single time needle aspiration [4], vacuum-assisted device application [7], and even creation of Eloesser's flap in rare cases.

Closed drainage combined with intravenous antibiotics rarely has been found to be successful as a standalone therapy in most reported cases but has been shown to have superior results in some reports [1–3, 8]; we reviewed those reports and found that most of the cases had pleural thickening <10 mm and short duration of symptoms (<7 days) excluding one case reported by Yauba et al. who had long duration of symptoms of 7 weeks.

In a case series review done by Akgül et al., 9 cases have been studied for approach to empyema necessitans. Tube drainage was the sole management in only 2 young patients (<30 years old) and 1 old patient (85 years old). In regard to diagnosis, 2 patients had tuberculous pleuritis and 1 patient was diagnosed with chronic pleuritis. In addressed patients, the pleural thickness was <10 mm and duration of symptoms was less than 7 days. There are no further details about radiological appearance.

Additionally, separate study done by Rosebush et al. showed success of tube drainage in a 4-week-old neonate who had diagnosis of pyogenic pleuritis complicated by empyema necessitans with 5 days history due to methicillin-resistant Staphylococcus aureus (MRSA). Pleural thickness was not reported in this case.

Moreover, reviewing the literature of previous cases of nontuberculous empyema necessitans, we found nearly 32 cases separated from the cases studied in the aforementioned case series by Akgül et al. and Rosebush et al.

Most of those cases had different microorganism etiologies. Reported organisms included *Streptococcus pneumoniae*, *methicillin-resistant Staphylococcus aureus, methicillin-sensitive Staphylococcus aureus*, *Klebsiella pneumoniae*, *Coccidioidomycosis*, *Porphyromonas gingivalis*, *COVID-19*, *Nocardia farcinica*, *Streptococcus agalactiae*, *Actinomyces meyeri*, *Aspergillus fumigatus*, *Bacteroides*, *Citrobacter freundii, Streptococcus constellatus*, *Pseudomonas*, *Actinomyces israelii*, *Aggregatibacter actinomycetemcomitans*, *Nocardia asteroids*, and *Streptococcus viridans*. Empyema necessitans caused by *Klebsiella pneumoniae* was found in 2 cases [9, 10]: first case was managed using surgical drainage (not specifying if done with radiological means or through video-assisted thoracoscopic surgery) and patient improved postdrainage but deteriorated later due to ventilator-associated pneumonia. The second case was managed through video-assisted thoracoscopic surgery; the patient had good outcome and discharged home on oral antibiotics. No more reported cases of empyema necessitans due to *Klebsiella pneumoniae* were found in the review due to rarity of this etiology.

Although variable options of treatment are available, each case must be tailored to specific treatment. After reviewing the literature, limited number of cases was found that reported management of empyema necessitans solely through pure closed drainage and IV antibiotics; approaching such cases requires maintaining high index of suspicion to rule out TB as primary cause if suggested by history and radiological exams, since there is a large proportion of reviewed cases that were diagnosed with tuberculous pleuritis [11].

In a study by Redden et al. [12], they compared surgical vs. nonsurgical methods for management of pleural empyema. They included 391 participants with different age groups from eight randomized controlled trials. There were 8 trials that were studied in this article; six studies reported no deaths in either treatment arm. Two studies reported mortality in adults aged over 18 years. Wait (1997) reported 1 death in each treatment arm. And another study (Bilgin 2006) reported no death in both arms. Both studies concluded that there is no difference in mortality between VATS and thoracostomy drainage, but the evidence was downgraded because of wide confidence intervals. Five studies compared mortality in children aged less than 18 years (Cobanoglu 2011; Kurt 2006; Marhuenda 2014; Peter 2009; Sonnappa 2006). No death is reported in both arms, but odds ratio (OR) was not estimable that explains why mortality was limited in this study. Furthermore, there was a significant difference in hospital stays between adult patients treated with video-assisted thoracoscopic surgery (VATS) and those who did not undergo surgery. Patients who underwent VATS had a shorter stay, with an average of 2.52 days compared to 4.10 days for nonsurgical patients. It is worth mentioning that included studies had controversial results in regard to total cost of treatment between VATS and surgical drainage arms in different reviewed studies by Redden et al.; the study concluded that the provided evidence is supporting both methods of treatment but not sufficient to change the practice.

In contrast to reviewed cases, our case had pleural thickening < 10 mm and short duration of symptoms (7 days) which could be an indicator for successful tube drainage as a sole treatment for empyema necessitans. Other factor that aided in the success of closed drainage is the large communication (1.4 cm) that exists between external and internal component found in CT and lack of septations which can allow for smooth drainage of external component through internal drainage. Facilitating drainage involves daily flushing of the pigtail and massaging the external component to aid its internal drainage, provided that there are no breaks in the integrity of the skin overlying the collection. Such breaks could introduce an external source of infection into the pleural space. Additionally, the empyema did not have solid material or heterogenicity on CT scan which ensures smooth drainage through small caliber catheter.

## 4. Conclusion

No unified management algorithm has been globally accepted for empyema necessitans. But reporting different management techniques in each case can encourage creating an algorithmic approach in the near future based on future case series study that can statistically identify the success rate of each intervention.

With proper selection of cases, complete resolution can be warranted through closed drainage and antibiotics with avoidance of surgical intervention which by results decreases hospital stay and avoidance of surgical and anesthesia complications.

We recommend closed drainage combined with IV antibiotics for cases of empyema necessitans with short duration of symptoms (less than 7 days) that has pleural thickening of <10 mm.

## Figures and Tables

**Table 1:** Pleural fluid analysis and biochemistry results.

Pleural fluid analysis	
Appearance	Turbid
Quantity	2 mL
Color	Yellowish
RBC (BF)	0.46
Polymorphic cells	64.8%
Monomorphic cells	35.4%
WBC (BF)	826.96

Biochemistry	
LDH	1452 U/L
Protein	67 g/L
pH	7
